# COVID-19 prevention at institutions of higher education, United States, 2020–2021: implementation of nonpharmaceutical interventions

**DOI:** 10.1186/s12889-023-15079-y

**Published:** 2023-01-24

**Authors:** Sarah Moreland, Nicole Zviedrite, Faruque Ahmed, Amra Uzicanin

**Affiliations:** 1 Centers for Disease Control and Prevention, 1600 Clifton Rd NE, Atlanta, GA 30329 USA; 2 Oak Ridge Institute for Science and Education, 1299 Bethel Valley Rd, Oak Ridge, TN 37830 USA

**Keywords:** COVID-19, Institutions of higher education, Nonpharmaceutical interventions, Pandemic response, Remote learning

## Abstract

**Background:**

In early 2020, following the start of the coronavirus disease 2019 (COVID-19) pandemic, institutions of higher education (IHEs) across the United States rapidly pivoted to online learning to reduce the risk of on-campus virus transmission. We explored IHEs’ use of this and other nonpharmaceutical interventions (NPIs) during the subsequent pandemic-affected academic year 2020–2021.

**Methods:**

From December 2020 to June 2021, we collected publicly available data from official webpages of 847 IHEs, including all public (*n* = 547) and a stratified random sample of private four-year institutions (*n* = 300). Abstracted data included NPIs deployed during the academic year such as changes to the calendar, learning environment, housing, common areas, and dining; COVID-19 testing; and facemask protocols. We performed weighted analysis to assess congruence with the October 29, 2020, US Centers for Disease Control and Prevention (CDC) guidance for IHEs. For IHEs offering ≥50% of courses in person, we used weighted multivariable linear regression to explore the association between IHE characteristics and the summated number of implemented NPIs.

**Results:**

Overall, 20% of IHEs implemented all CDC-recommended NPIs. The most frequently utilized NPI was learning environment changes (91%), practiced as one or more of the following modalities: distance or hybrid learning opportunities (98%), 6-ft spacing (60%), and reduced class sizes (51%). Additionally, 88% of IHEs specified facemask protocols, 78% physically changed common areas, and 67% offered COVID-19 testing. Among the 33% of IHEs offering ≥50% of courses in person, having < 1000 students was associated with having implemented fewer NPIs than IHEs with ≥1000 students.

**Conclusions:**

Only 1 in 5 IHEs implemented all CDC recommendations, while a majority implemented a subset, most commonly changes to the classroom, facemask protocols, and COVID-19 testing. IHE enrollment size and location were associated with degree of NPI implementation. Additional research is needed to assess adherence to NPI implementation in IHE settings.

**Supplementary Information:**

The online version contains supplementary material available at 10.1186/s12889-023-15079-y.

## Background

In early 2020, nonpharmaceutical interventions (NPIs) were implemented across the United States (US) to slow the spread of severe acute respiratory syndrome coronavirus 2 (SARS CoV-2), the causative agent of coronavirus disease 2019 (COVID-19). These included mass gathering cancelations, travel restrictions, use of facemasks, and physical distancing, including a pivot to distance learning for both K-12 schools and institutions of higher education (IHEs) [1, 2].

By August 2020, COVID-19 incidence was highest among young adults 20–29 years of age [1]. While generally at lower risk for severe disease and outcomes than older age groups, infected young adults transmit infection to others in their communities [3, 4]. Simulation models suggest that the introduction of university students into a population worsened COVID-19 outcomes in the broader community between August 15, 2020, and December 31, 2020 [5]. However, experiences from the US and Taiwan suggest that the safe re-opening of IHEs may be feasible with a combination of containment and mitigation strategies [6–9].

The rapid, near-universal transition to distance learning experienced by US IHEs in March 2020 was unprecedented in terms of duration and nationwide scale, thereby leaving administrators, faculty, and students without a clear path to return to on-campus operations [2]. In May 2020, CDC issued guidance for IHE operations during the coming academic year, 2020–2021, that was updated regularly [10, 11].

We assessed how IHEs adapted educational instruction and other processes during the 2020–2021 academic year and how they implemented recommended NPIs to prevent on-campus SARS-CoV-2 transmission.

## Methods

### Study sample

From the National Center for Education Statistic’s (NCES) Integrated Postsecondary Education Data System (IPEDS) [12], we identified traditional 4-year undergraduate public and private IHEs within the 50 states and DC, excluding primarily graduate, clinical, or trade programs. Data were obtained for the 2018–2019 academic year, the most recent year of data prior to the COVID-19 pandemic. All 547 public and a stratified random sample of private IHEs meeting inclusion criteria were included in the study. The universe of 1181 private IHEs was stratified by student enrollment number as defined by IPEDS (< 1000, 1000 - 4999, 5000 - 9999, 10,000 - 19,999, ≥ 20,000). Based on sample size calculations using a 95% confidence interval with a 5% margin of error, 26% of eligible private IHEs were randomly selected within each stratum (97 of 398 private IHEs, 161 of 625 private IHEs, 24 of 93 private IHEs, 12 of 47 private IHEs, and 6 of 18 private IHEs, respectively).

### Data collection

From December 2020 through June 2021, we conducted searches of publicly available online data from IHE-run websites, including those pertaining to COVID-19 response, plans to return to in-person learning, and campus announcements, to examine NPIs implemented within 547 public IHEs and the sample of 300 private IHEs in response to the ongoing COVID-19 pandemic. From IHE websites, a team of eight data collectors sought available information about NPIs implemented, with particular attention to those recommended in the October 29, 2020 update to the US Centers for Disease Control and Prevention’s (CDC) Guidance for IHEs [11]. We summarized the CDC guidance into seven broad categories of interventions and designed a standardized data collection form, including fields for changes to the academic calendar, learning environment, residence halls, common spaces, and student dining; campus COVID-19 testing protocols; and facemask requirements. We collected data on NPIs specifically related to the COVID-19 pandemic rather than those routinely recommended for everyday use regardless of pandemic status, such as hand washing, cleaning, and respiratory etiquette [13]. Where available, we documented how each NPI was implemented and the primary learning format utilized by the IHE. Table 1 lists all NPIs surveyed and how they were classified. Data collection for public and private IHEs was done consecutively, December 2020 – May 2021 and April 2021 – June 2021, respectively.

**Table 1:** NPIs^ab^ abstracted from IHE^b^ websites

Category	Variable	Variable Type	Response Examples
1. Academic Calendar	Has the academic calendar been changed in response to COVID-19?	Yes, No, or Not found	
	How was each campus break (Thanksgiving, winter break, spring break, etc.) changed?	Select all that apply	Shortened, lengthened, eliminated, unchanged
2. Learning Environment	Has the learning environment been adapted in response to COVID-19?	Yes, No, or Not found	
	How has the learning environment been changed?	Select all that apply	Spacing between seats, hybrid attendance, reduced class size, other
	In what format(s) are classes being offered?	Select all that apply	In person, hybrid, distance learning
3. On-Campus Housing	Has the capacity of residence halls been changed or reduced?	Yes, No, Not found, or Does not apply	
	How has residence hall capacity been changed?	Select all that apply	Single-occupancy rooms, reduced capacity rooms, designated isolation spaces, other
	Have residence hall guest and visitor policies been changed?	Yes, No, Not found, or Does not apply	
	How have residence hall guest policies been changed?	Select all that apply	Eliminate visitation, limit number of visitors, restrict visitors based on residence, other
4. Campus Common Spaces	Have common spaces been physically changed or restricted?	Yes, No, or Not found	
	How have common spaces been changed?	Select all that apply	Removal of furniture, signage and floor stickers, closure of spaces, limit capacity, other
5. Campus Dining Services	Have dining halls and services been changed or limited?	Yes, No, or Not found	
	How have dining services been changed?	Select all that apply	Limit capacity, to-go dining, installation of partitions, removal of self-serve options, other
6. COVID-19 Testing	Does the IHE offer COVID-19 testing on campus?	Yes, No, or Not found	
	Who is eligible to utilize campus COVID-19 testing services?	Select all that apply	Residential students, all students, staff, non-campus affiliates
	What criteria must be met to receive a COVID-19 test?	Select all that apply	Asymptomatic, suspected contact, confirmed contact, symptomatic, no criteria
	When, if ever, is COVID-19 testing required?	Select all that apply	Surveillance testing, upon arrival for the semester
	How did COVID-19 testing availability change between semesters?	More available, Less available, No change	
7. Facemask Requirements	Where are facemasks required on campus?	Select all that apply	Indoor, outdoor (where 6 ft. cannot be maintained)
	Who do facemask requirements apply to?	Select all that apply	Students, staff, visitors

^a^ NPIs derived from October 29, 2020 update of US Centers for Disease Control and Prevention Guidance for IHEs

^b^ NPIs, nonpharmaceutical interventions; IHEs, institutions of higher education

### Final dataset

**Fig. 1:**
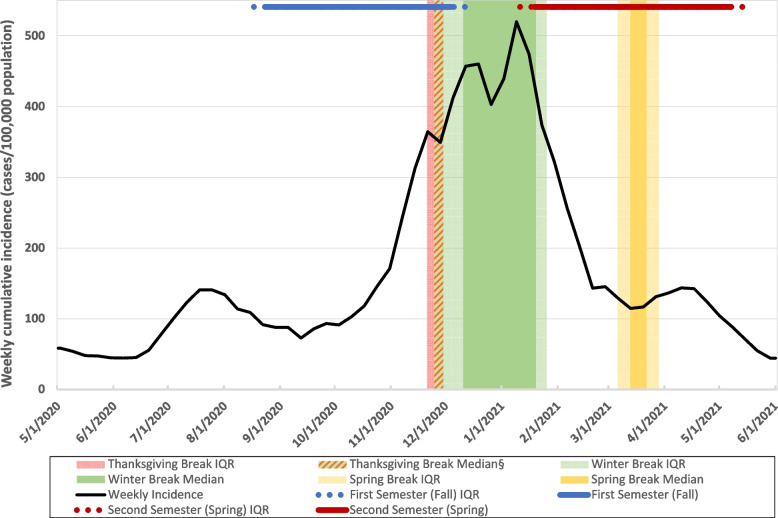
Timeline of IHE* academic calendars† and weekly national COVID-19 incidence‡. * IHE, institution of higher education. † 2020–2021 Academic calendar dates collected from each IHE website. Median (50th percentile) and IQR (25th and 75th percentile) were calculated for milestone dates. ‡ Weekly Incidence (cases per week per 100,000 people) derived from JHU CSSE COVID-19 Data and U.S. Census Bureau 2020 population estimation. § Thanksgiving median and IQR, and winter break IQR dates encompass November 25–29, 2020

From the NCES IPEDS dataset, we collected data on the characteristics of IHEs included in our study, including affiliation (public vs. private), student enrollment, degree of urbanization, and location information (county name and code). IHEs were categorized into four geographic regions as defined by the US Census Bureau [14]. IHEs were further categorized by enrollment size into small (> 1000 students), medium (1000–9999 students), and large (≥10,000 students). We obtained publicly available county-level data on COVID-19 cases from January 2020 – June 2021 from the COVID-19 Data Repository by the Center for Systems Science and Engineering (CSSE) at Johns Hopkins University [15, 16]. The national incidence rate per 100,000 population (CI) for each week during the period May 2020 – June 2021 was calculated using the summated reported daily COVID-19 case counts and the US Census Bureau 2020 estimated residential population [15–17]. To visualize national COVID-19 trends in the context of the 2020–2021 academic year, IHE academic calendar dates were collected and summarized by median and IQR dates for semester start and end, and each break within the first semester, classified as August through December 2020, and the second semester, classified as January through May 2021, and illustrated graphically along with US weekly COVID-19 incidence (Fig. 1). To simplify variation in calendar structure between IHEs, our analysis was restricted to dates encompassing the common two-semester academic year, excluding summer or other abbreviated terms.

### Analysis and visualization

Descriptive statistics were calculated to summarize characteristics of the sampled IHEs, including affiliation, enrollment, US Census Bureau Region, and urbanicity. The number of NPIs implemented was summated to create a score from 0 to 7, representing a level of compliance with the seven categories of NPIs shown in Table 1 [11]. For each NPI category, a score of 1 was assigned if it was implemented through at least one modality, such as the examples listed in Table 1, and a score of 0 was assigned if it was not implemented or if implementation was unknown. To generalize findings to all US IHEs, we performed weighted analysis to assess congruence with the guidance. Sampling weights representing the inverse of the sampling fractions were used to account for the unequal probability of selection into the sample [18]. For IHEs that offered ≥50% courses in-person, we used weighted multivariable linear regression to explore the associations between IHE characteristics and the summated number of implemented NPIs. Weighted multivariable logistic regression was performed to explore associations between IHE characteristics and individual NPIs among IHEs offering ≥50% courses in person.

Analysis was conducted using SAS 9.4. Figures were created in Tableau, Microsoft Power BI, and Microsoft Excel.

## Results

### Characteristics of the study sample

A total of 847 IHEs were sampled from the universe of 1728 IHEs, including all 547 (32%) public and 300 of the 1181 (68%) private institutions (Table 2). Of the four US Census Bureau regions, the South contained the highest number of IHEs, and the West contained the fewest. Half of the sampled IHEs were in cities, and the fewest were in rural areas. IHEs were categorized based on student enrollment: 24% enrolled under 1000 students, 44% enrolled 1000–4999 students, 13% enrolled 5000–9999 students, 10% enrolled 10,000–19,999 students, and 9% enrolled 20,000 or more students.

**Table 2:** Characteristics of sampled IHEs^a^ by enrollment size^b^

Enrollment size^b^	Total	Under 1000 students	1000–4999 students	5000–9999 students	10,000–19,999 students	20,000 students and above	***p***-value‡
Number of IHEs, n (%)^c^	847 (100)	118 (24)	302 (44)	152 (13)	139 (10)	136 (9)	
Affiliation, n (%)^c^							< 0.0001
Public	547 (32)	21 (5)	141 (18)	128 (58)	127 (73)	130 (88)	
Private	300 (68)	97 (95)	161 (82)	24 (42)	12 (27)	6 (12)	
United States Census Region, n (%)^c^							< 0.0001
Northeast	204 (26)	38 (25)	79 (28)	45 (35)	29 (23)	13 (13)	
Midwest	213 (28)	33 (32)	80 (30)	35 (24)	37 (26)	28 (19)	
South	305 (34)	35 (33)	112 (34)	53 (29)	50 (35)	55 (39)	
West	125 (12)	12 (11)	31 (8)	19 (13)	23 (15)	40 (30)	
Urbanicity^d^, n (%)^c^							< 0.0001
Rural	50 (7)	18 (14)	29 (8)	3 (1)	0 (0)	0 (0)	
Town	186 (18)	18 (15)	89 (22)	42 (20)	29 (17)	8 (5)	
Suburban	194 (25)	33 (25)	69 (26)	49 (31)	29 (20)	23 (16)	
City	417 (50)	49 (45)	115 (44)	67 (47)	81 (63)	105 (79)	

^a^ IHEs, institutions of higher education

^b^ Enrollment size based on total students enrolled for credit as reported by the Integrated Postsecondary Education Data System

‡ *P* value assesses whether enrollment size differs by the characteristics

^c^ The universe of 1181 private IHEs was stratified by enrollment size. Based on sample size calculations using a 95% confidence interval with a 5% margin of error, 26% of eligible private IHEs were randomly selected within each stratum (< 1000: 97 of 398 private IHEs, 1000 – 4999: 161 of 625 private IHEs, 5000 – 9999: 24 of 93 private IHEs, 10,000 – 19,999: 12 of 47 private IHEs, and ≥ 20,000: 6 of 18 private IHEs). Weights represent the inverse of the sampling fractions. n (%) represents the unweighted number (n) and the weighted percent of the universe of 547 public and 1181 private IHEs

^d^ Level of urbanization based on urban boundaries by Census GEO as reported by the Integrated Postsecondary Education Data System

### CDC guidance compliance

**Fig. 2:**
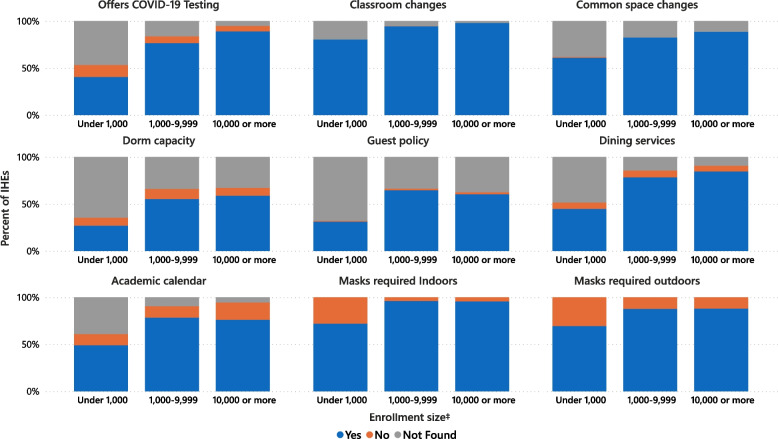
NPIs* implemented† by IHEs* in response to the COVID-19 pandemic by enrollment size‡ * NPIs, nonpharmaceutical interventions; IHEs, institutions of high education. † NPIs derived from CDC Guidance October 29, 2020 update of US Centers for Disease Control and Prevention Guidance for IHEs. ‡ Enrollment size based on total students enrolled for credit as reported by the Integrated Postsecondary Education Data System

The October 29, 2020, CDC Guidance for IHEs was comprised of 7 broad interventions [11], and their implementation resulted in at least 24 distinct modalities in practice, summarized in Table 3. All recommended NPIs were addressed and implemented through at least one modality by 20% of IHEs, and 4% implemented none (Table 3). IHEs implementing all 7 NPIs more often had higher enrollment, while those implementing none most often had < 1000 students. The four-NPI combination of changes to the learning environment, common spaces, dining, and facemask protocols were implemented by 62% of IHEs. Figure 2 illustrates the implementation of NPIs by enrollment size.

**Table 3:** NPIs^ab^ implemented by IHEs^a^ in response to the COVID-19 pandemic by enrollment size ^c^

Enrollment size﻿^**c**^	Total	Under 1000 students	1000–4999 students	5000–9999 students	10,000–19,999 students	20,000 students and above
Number of IHEs, n (%)^d^	847 (100)	118 (24)	302 (44)	152 (13)	139 (10)	136 (9)
Selected combinations of NPIs implemented by IHEs, n (%)^d^						
7 Nonpharmaceutical interventions						
All surveyed CDC guidelines^**b**^	182 (20)	9 (7)	70 (24)	38 (25)	27 (24)	38 (28)
5 Nonpharmaceutical interventions						
COVID-19 testing, masks, changes to classroom, common space, and dining	465 (49)	27 (21)	160 (55)	89 (56)	92 (68)	97 (70)
Masks, changes to academic calendar, classroom, common space, and dining	433 (47)	30 (25)	159 (52)	84 (56)	82 (62)	78 (55)
4 Nonpharmaceutical interventions						
Masks, changes to classroom, common space, and dining	557 (62)	41 (35)	204 (70)	104 (67)	108 (79)	100 (72)
0 Nonpharmaceutical interventions	19 (4)	15 (14)	4 (2)	0 (0)	0 (0)	0 (0)
Primary format of course delivery, n (%)^d^						
Primary format identified	393 (52)	48 (53)	138 (52)	72 (53)	68 (56)	67 (48)
Mostly distance learning (< 50% in person)	166 (38)	21 (42)	37 (27)	32 (39)	35 (53)	41 (58)
Balanced (50% in person and 50% distance learning)	153 (36)	16 (32)	58 (37)	32 (44)	27 (38)	20 (31)
Mostly in person learning (> 50% in person)	74 (26)	11 (26)	43 (36)	8 (17)	6 (9)	6 (11)
Changes in class delivery between academic terms						
More distance learning	27 (5)	5 (6)	8 (4)	5 (4)	3 (3)	6 (8)
No change	164 (39)	20 (43)	51 (36)	34 (46)	29 (15)	30 (42)
More in person learning	55 (17)	7 (16)	20 (18)	11 (20)	8 (15)	9 (15)
Not found	147 (39)	16 (34)	59 (43)	22 (30)	28 (48)	22 (34)
Primary format not identified	401 (48)	47 (47)	144 (48)	75 (47)	67 (44)	68 (52)
1. Made changes to Academic Calendar, n (%)^d^						
Yes	623 (67)	58 (42)	236 (73)	120 (78)	101 (76)	108 (77)
Specific changes to academic breaks (where changes are not unknown)						
Lengthening of Thanksgiving Break (*n* = 418)	323 (77)	42 (87)	119 (78)	64 (72)	43 (75)	54 (70)
Does not return to in-person learning after Thanksgiving break	315 (76)	42 (82)	116 (76)	63 (72)	41 (75)	53 (68)
Lengthening of Winter Break (*n* = 386)	200 (51)	27 (53)	65 (46)	43 (60)	33 (51)	32 (56)
Elimination of Spring Break (*n* = 511)	341 (69)	35 (71)	131 (70)	75 (77)	49 (66)	51 (58)
No	120 (14)	14 (14)	32 (12)	23 (16)	29 (17)	22 (16)
Unknown	104 (19)	46 (44)	34 (15)	9 (6)	9 (7)	6 (7)
2. Made changes to learning environment, n (%)^d^						
Yes	794 (91)	95 (78)	282 (93)	147 (96)	135 (96)	135 (99)
Offered distance or hybrid learning	783 (98)	91 (95)	278 (98)	144 (96)	135 (100)	135 (100)
Physical changes to the classroom						
Space seating 6 ft. apart	465 (60)	43 (48)	177 (66)	92 (65)	81 (57)	72 (54)
Reduced class size	413 (51)	39 (41)	150 (53)	83 (54)	74 (57)	67 (50)
Alternating attendance of students in classes	297 (38)	27 (28)	119 (43)	48 (32)	59 (49)	44 (33)
No	1 (0)	0 (0)	1 (0)	0 (0)	0 (0)	0 (0)
Unknown	52 (9)	23 (22)	19 (7)	5 (4)	4 (4)	1 (1)
3. Made changes to Student Housing, n (%)^d^						
Residential housing capacity						
Yes	445 (50)	32 (28)	157 (54)	94 (59)	82 (62)	80 (58)
Reduced housing density and/or capacity	228 (55)	14 (44)	83 (57)	47 (55)	42 (58)	42 (53)
Single occupancy rooms	108 (20)	6 (19)	34 (17)	22 (19)	22 (26)	24 (30)
No	82 (11)	10 (10)	35 (12)	14 (13)	11 (8)	12 (9)
Unknown	301 (36)	69 (57)	105 (32)	42 (27)	42 (27)	43 (32)
IHE does not offer student housing	19 (3)	7 (5)	5 (2)	2 (1)	4 (3)	1 (1)
Guest policy						
Yes	497 (58)	37 (32)	198 (69)	96 (66)	80 (61)	86 (64)
Did not allow guests in living areas	257 (50)	16 (45)	99 (49)	50 (50)	41 (50)	51 (61)
Limited number of guests	120 (22)	7 (19)	47 (22)	25 (30)	19 (18)	22 (26)
No	14 (2)	1 (1)	6 (2)	1 (0)	5 (5)	1 (1)
Unknown	317 (37)	73 (62)	93 (27)	53 (33)	5 (31)	48 (34)
IHE does not offer student housing	19 (3)	7 (5)	5 (2)	2 (1)	4 (3)	1 (1)
4. Made changes to campus common areas, n (%)^d^						
Yes	691 (78)	72 (59)	248 (83)	127 (82)	128 (94)	116 (84)
Restricted room or area capacity	508 (74)	51 (74)	187 (75)	97 (76)	99 (79)	74 (63)
Use of physical guides (signage, floor stickers, etc.)	428 (58)	36 (50)	150 (56)	89 (70)	78 (59)	75 (65)
Use of physical barriers (furniture removal, partitions, etc.)	397 (54)	30 (44)	139 (54)	79 (61)	76 (56)	73 (64)
No	1 (0)	1 (1)	0 (0)	0 (0)	0 (0)	0 (0)
Unknown	155 (22)	45 (40)	54 (17)	25 (18)	11 (6)	20 (16)
5. Made changes to campus dining halls, n (%)^d^						
Yes	642 (72)	53 (44)	234 (80)	122 (80)	119 (89)	114 (84)
Increased grab and go options	461 (73)	40 (74)	169 (73)	87 (70)	78 (68)	87 (75)
Reduced capacity for indoor seating	404 (67)	32 (60)	158 (70)	77 (65)	75 (71)	62 (56)
Physical barriers or guides in lines and seating areas	308 (45)	24 (44)	110 (42)	56 (48)	55 (47)	63 (54)
Elimination of self service	298 (48)	28 (53)	128 (55)	47 (33)	52 (47)	43 (35)
No	58 (3)	8 (2)	19 (2)	14 (6)	8 (5)	9 (6)
Unknown	147 (25)	57 (54)	49 (18)	16 (14)	12 (6)	13 (10)
6. Offered on-campus COVID-19 testing, n (%)^d^						
Yes	641 (67)	48 (32)	233 (73)	125 (82)	116 (85)	129 (95)
Population eligible for COVID-19 testing						
Residential students	576 (90)	39 (77)	198 (90)	112 (93)	104 (92)	123 (96)
All students	543 (85)	38 (74)	181 (84)	107 (90)	100 (87)	117 (91)
Staff	400 (61)	21 (45)	12 (61)	81 (61)	79 (65)	92 (72)
No minimum criteria to be tested	399 (62)	25 (39)	141 (66)	81 (68)	55 (58)	86 (67)
Scenarios when testing is required	495 (83)	43 (85)	190 (88)	94 (80)	72 (70)	96 (77)
Upon arrival for the semester	363 (60)	32 (62)	155 (71)	74 (54)	44 (38)	56 (43)
Random or scheduled surveillance testing of all students	271 (47)	21 (32)	104 (51)	52 (53)	33 (34)	61 (50)
Random or scheduled surveillance testing of residential students	129 (20)	2 (4)	38 (21)	27 (21)	24 (20)	38 (31)
Change in testing availability between academic terms						
More available	203 (32)	19 (30)	69 (32)	43 (40)	31 (27)	41 (33)
Less available	4 (0)	0 (0)	0 (0)	3 (2)	1 (1)	0 (0)
No change	106 (16)	9 (23)	36 (16)	22 (15)	21 (18)	18 (13)
Unknown	328 (51)	20 (47)	118 (51)	57 (43)	63 (54)	70 (54)
No	64 (9)	15 (14)	20 (7)	13 (8)	12 (9)	4 (3)
Unknown	142 (24)	55 (54)	59 (20)	14 (10)	11 (6)	3 (2)
7. Required masks or face coverings, n (%)^d^						
Indoors	784 (88)	85 (68)	288 (95)	148 (96)	133 (95)	130 (96)
Students	781 (88)	83 (69)	287 (95)	148 (96)	133 (95)	130 (96)
Staff	781 (88)	84 (67)	287 (94)	148 (96)	132 (94)	130 (96)
Visitors	750 (85)	82 (65)	275 (92)	137 (91)	128 (92)	128 (95)
Outdoors	723 (82)	82 (65)	262 (88)	137 (88)	120 (87)	122 (89)
Students	721 (82)	81 (64)	261 (87)	137 (88)	120 (87)	122 (89)
Staff	721 (82)	82 (65)	260 (87)	137 (88)	120 (87)	122 (89)
Visitors	694 (80)	80 (63)	252 (86)	126 (83)	117 (86)	119 (87)

^a^ NPIs, nonpharmaceutical interventions; IHEs, institutions of higher education

^b^ NPIs derived from October 29, 2020 update of US Centers for Disease Control and Prevention Guidance for IHEs. Numbered items indicate each of the 7 broad NPIs being surveyed, followed by common modalities in which each NPI was implemented

^c^ Enrollment size based on total students enrolled for credit as reported by the Integrated Postsecondary Education Data System

^d^ The universe of 1181 private IHEs was stratified by enrollment size. Based on sample size calculations using a 95% confidence interval with a 5% margin of error, 26% of eligible private IHEs were randomly selected within each stratum (< 1000: 97 of 398 private IHEs, 1000 – 4999: 161 of 625 private IHEs, 5000 – 9999: 24 of 93 private IHEs, 10,000 – 19,999: 12 of 47 private IHEs, and ≥ 20,000: 6 of 18 private IHEs). Weights represent the inverse of the sampling fractions. n (%) represents the unweighted number (n) and the weighted percent of the universe of 547 public and 1181 private IHEs

### Primary learning method

**Fig. 3:**
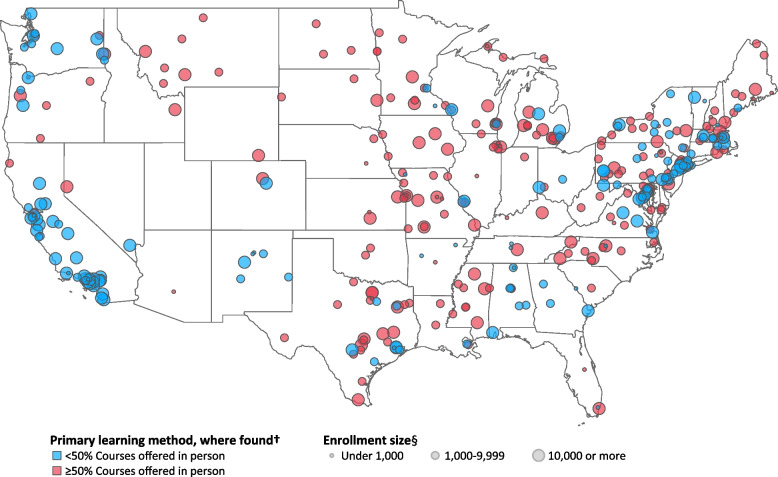
Geographical distribution* of primary learning method† offered IHEs‡, by enrollment size § * Geographical distribution of primary learning method with size of the point representing institution enrollment size category. Generated with Tableau Software. † 227 IHEs offer courses ≥50% in person. One hundred sixty-six IHEs offer courses < 50% in person. Primary learning method not found for 454 IHEs. ‡ IHEs, institutions of higher education. § Enrollment size based on total students enrolled for credit as reported by the Integrated Postsecondary Education Data System

**Fig. 4:**
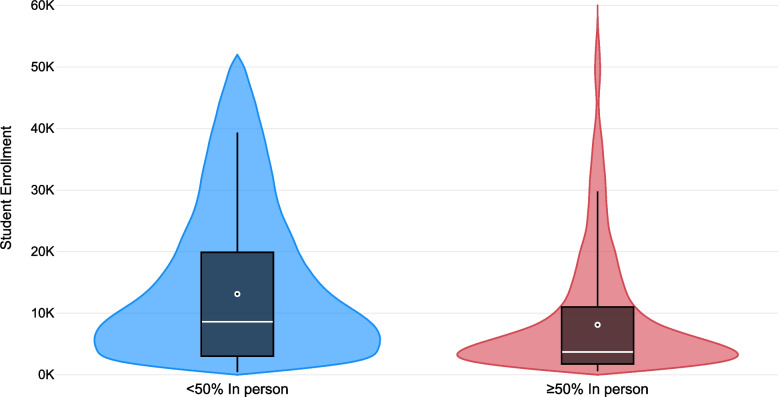
Distribution* of IHE† student enrollment by primary course format ‡ offered by IHEs * Violin plot of student enrollment kernel density distribution within primary learning formats. Boxplot dot and line represent the mean and median enrollment, respectively. Generated in Microsoft Power BI. † IHEs, institutions of higher education. ‡ 227 IHEs offer courses ≥50% in person. One hundred sixty-six IHEs offer courses < 50% in person. Primary learning method not found for 454 IHEs

The primary format of course delivery was identified for 393 IHEs, of which 38% offered courses < 50% in person, 26% offered courses > 50% in person, and 36% offered courses roughly 50% through distance learning and 50% in person (Table 3). IHEs that offered ≥50% of courses in person were most often smaller by student enrollment and in the Midwest (Figs. 3 and 4). In multivariable regression, IHEs having 1000–4999 students enrolled versus < 1000, being in the Midwest versus the Northeast, and being in a town versus urban were more likely to offer ≥50% of courses in person (Table 4). IHEs in the West versus Northeast were less likely to have offered ≥50% of courses in person.

**Table 4:** Characteristics of IHEs^a^ offering primarily in-person learning^b^ versus primarily distance learning

IHE Characteristics	Learns ≥ 50% in person, n (%)^c^	Learns < 50% in person, n (%)^c^	*p*-value	Multivariable Analysis^d^			
				AOR^**a**^	95% Confidence Limits		***p***-value
					Lower	Upper	
All IHEs where primary learning method^b^ was identified	227 (62)	166 (38)					
Affiliation							
Public	129 (52)	118 (48)	**< 0.0001**	0.66	0.41	1.06	0.088
Private	98 (67)	48 (33)	–	1 (Referent)	–	–	–
Enrollment size^e^							
Under 1000 students	27 (58)	21 (42)	–	1 (Referent)	–	–	–
1000–4999 students	101 (73)	37 (27)	**< 0.0001**	**2.08**	1.02	4.25	**0.020**
5000–9999 students	40 (61)	32 (39)	0.328	1.52	0.66	3.50	0.450
10,000–19,999 students	33 (47)	35 (53)	0.059	0.88	0.38	2.04	0.077
20,000 students and above	26 (42)	41 (58)	**< 0.0001**	1.30	0.59	2.87	0.994
United States Census Region							
Northeast	56 (58)	52 (42)	–	1 (Referent)	–	–	–
Midwest	79 (91)	11 (9)	**< 0.0001**	**7.61**	3.47	16.70	**< 0.0001**
South	66 (58)	41 (42)	0.178	1.05	0.58	1.89	0.176
West	26 (33)	62 (67)	**< 0.0001**	**0.43**	0.23	0.81	**< 0.0001**
Degree of urbanicity^f^							
Rural	15 (60)	8 (40)	0.499	0.99	0.35	2.784	0.382
Town	67 (81)	18 (19)	**0.0003**	**2.88**	1.45	5.74	**0.006**
Suburban	44 (59)	44 (41)	0.161	1.27	0.72	2.238	0.720
Urban	101 (58)	96 (42)	–	1 (Referent)	–	–	–

^a^ IHEs, institutions of high education; AOR, adjusted odds ratio

^b^ 227 IHEs offer courses ≥50% in person. 166 IHEs offer courses < 50% in person. Primary learning method not found for 454 IHEs

^c^ The universe of 1181 private IHEs was stratified by enrollment size. Based on sample size calculations using a 95% confidence interval with a 5% margin of error, 26% of eligible private IHEs were randomly selected within each stratum (< 1000: 97 of 398 private IHEs, 1000 – 4999: 161 of 625 private IHEs, 5000 – 9999: 24 of 93 private IHEs, 10,000 – 19,999: 12 of 47 private IHEs, and ≥ 20,000: 6 of 18 private IHEs). Weights represent the inverse of the sampling fractions. n (%) represents the unweighted number (n) and the weighted percent of the universe of 547 public and 1181 private IHEs

^d^ Multivariable logistic regression analysis of IHE characteristics with primary form of course delivery as the dependent variable (0 = Learns < 50% in person, 1 = Learns ≥50% in person)

^e^ Enrollment size based on total students enrolled for credit as reported by the Integrated Postsecondary Education Data System

^f^ Level of urbanization based on urban boundaries by Census GEO as reported by the Integrated Postsecondary Education Data System

**Fig. 5:**
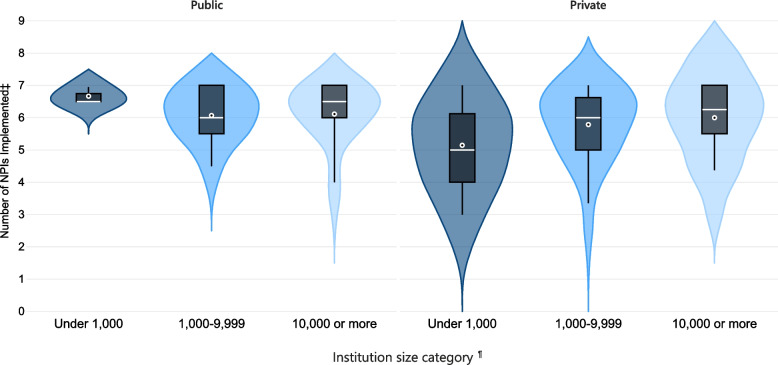
Distribution* of number of NPIs† implemented† by IHEs† learning ≥50% in person§ by type and size¶. * Violin plot of total NPIs implemented kernel density distribution within enrollment size categories. Boxplot dot and line represent the mean and median number of NPIs implemented within each category, respectively. Generated in Microsoft Power BI. † NPIs, nonpharmaceutical interventions; IHEs, institutions of high education. ‡ NPIs derived from October 29, 2020 update of US Centers for Disease Control and Prevention Guidance for IHEs and summated ranging 0–7. § 227 IHEs offer courses ≥50% in person. One hundred sixty-six IHEs offer courses < 50% in person. Primary learning method not found for 454 IHEs. ¶ Enrollment size based on total students enrolled for credit as reported by the Integrated Postsecondary Education Data System

Among the subset of IHEs that reported offering ≥50% courses in person, the number of NPIs implemented was higher among IHEs in the Northeast compared to the other regions, though only significantly compared to the Midwest and West, and among IHEs having 1000–4999 and 10,000–19,999 students than those with < 1000 students (Table 5). When examined by affiliation, private IHEs (95% CI: [5.40, 5.87]) exhibited wider variation in the number of NPIs implemented than public IHEs (95% CI: [5.97, 6.23]), most notably in IHEs with under 1000 students (Table 5, Fig. 5).

**Table 5:** IHE^a^ characteristics associated with compliance^b^ with CDC Guidance^c^ among IHEs offering primarily in-person learning

IHE Characteristics	Number of IHEs (Unweighted n)	Weighted^d^ mean score for compliance^**b**^ with CDC guidance^c^	95% Confidence Limits		***p***-value	Multivariable Analysis^e^			
			Lower	Upper		Parameter Estimate Beta	95% Confidence Limits		***p***-value
							Lower	Upper	
All IHEs offering ≥50% courses in person	227	5.75	5.57	5.93					
Affiliation									
Public	129	6.10	5.97	6.23	**0.001**	0.23	−0.15	0.60	0.234
Private	98	5.64	5.40	5.87	–	0 (Referent)	–	–	–
Enrollment size^f^									
Under 1000 students	27	5.19	4.65	5.73	–	0 (Referent)	–	–	–
1000–4999 students	101	5.82	5.58	6.07	**0.032**	**0.56**	0.01	1.11	**0.048**
5000–9999 students	40	5.95	5.50	6.39	**0.031**	0.65	−0.10	1.39	0.087
10,000–19,999 students	33	6.18	5.80	6.55	**0.002**	**0.84**	0.21	1.47	**0.009**
Over 20,000 students	26	5.97	5.30	6.64	0.064	0.71	−0.14	1.56	0.102
United States Census Region									
Northeast	56	6.17	5.83	6.51	–	0 (Referent)	–	–	–
Midwest	79	5.62	5.35	5.89	**0.012**	**−0.57**	−0.98	−0.16	**0.006**
South	66	5.77	5.35	6.20	0.124	−0.48	−0.99	0.04	0.072
West	26	5.19	4.54	5.84	**0.007**	**−1.07**	−1.75	−0.39	**0.002**
Degree of urbanicity^g^									
Rural	15	6.15	5.57	6.73	0.170	0.16	−0.47	0.80	0.615
Town	67	5.88	5.55	6.21	0.511	0.05	−0.37	0.47	0.799
Suburban	44	5.53	5.14	5.92	0.412	−0.35	−0.81	0.10	0.129
Urban	101	5.74	5.45	6.02	–	0 (Referent)	–	–	–

^a^ IHEs, institutions of higher education

^b^ Compliance with CDC guidance represented by the summating number of NPIs implemented, as derived from CDC guidance, by the IHE into a score from 0 to a maximum of 7

^c^ October 29, 2020 update of US Centers for Disease Control and Prevention Guidance for IHEs

^d^ The universe of 1181 private IHEs was stratified by enrollment size. Based on sample size calculations using a 95% confidence interval with a 5% margin of error, 26% of eligible private IHEs were randomly selected within each stratum (< 1000: 97 of 398 private IHEs, 1000 – 4999: 161 of 625 private IHEs, 5000 – 9999: 24 of 93 private IHEs, 10,000 – 19,999: 12 of 47 private IHEs, and ≥ 20,000: 6 of 18 private IHEs). Weights represent the inverse of the sampling fractions

^e^ Multivariable linear regression analysis of IHE characteristics with compliance with CDC Guidance as to the dependent variable, represented by the summating number of NPIs implemented by the IHE into a score of 0–7. Parameter estimate beta represents the predicted change in compliance score compared to that of the reference group holding all other characteristics constant

^f^ Enrollment size based on total students enrolled for credit as reported by the Integrated Postsecondary Education Data System

^g^ Level of urbanization based on urban boundaries by Census GEO as reported by the Integrated Postsecondary Education Data System

### Academic calendar

Over two-thirds of IHEs made changes to their academic calendars in response to COVID-19 (Table 3). Figure 1 summarizes common IHE academic breaks. Among the 418 IHEs with available information, 77% lengthened Thanksgiving break, nearly all of which extended it into winter break and did not return to in-person instruction for the remainder of the calendar year. Where identified (386 IHEs), half of IHEs specifically lengthened winter break with additional days off, a period of distance learning, or a combination of the two. Among the 511 IHEs where information was identified, 68% eliminated spring break.

### Learning environment

The most frequently utilized NPI, implemented by 91% of IHEs, was changes to the learning environment. The changes made most often among this subset included one or more of the following: increased availability of distance or hybrid learning options (98%), spaced desks and classroom seating 6-ft apart (60%), reduced classroom capacity (51%), and alternated classroom attendance schedules (38%) (Table 3). Only one IHE explicitly did not implement any changes, and information was not identified for 9%.

### Housing

Residential housing capacity policies were changed in 50% of IHEs, not changed in 11%, and not identified in 36% (Table 3). On-campus housing was not offered in 3%. Overall housing capacity or density was reduced in 55% of IHEs, and, notably, dorms were limited to single occupancy in 20%. Residential housing guest policies were changed in 58% of IHEs, not changed in 2%, and not identified in 37% (Table 3).

### Common spaces

Changes to common campus spaces were made by 78% of IHEs, practiced most often among this subset as one or more of the following: reduced capacity (74%), physical guides (58%), such as signs or floor markers, or physical modifications (54%), such as furniture removal or use of partitions (Table 3). Changes were most often made by IHEs with ≥1000 students. Only one IHE explicitly did not implement any changes to common spaces, and information was not identified for 22% IHEs.

### Dining services

Dining services were modified by 72% of IHEs, among which most implemented one or more of the following methods: increased to-go meal options (73%), reduced indoor seating capacity (67%), and physical modifications, such as furniture removal or use of partitions (45%) or elimination of self-service options (48%) (Table 3). IHEs with ≥1000 students made changes to dining most often. Dining services were explicitly unchanged in 3% of IHEs, and information was not identified for 25%.

### COVID-19 testing

Over two-thirds of IHEs were found to offer on-campus COVID-19 testing, most often larger IHEs (Table 3). Among IHEs that offered on-campus COVID-19 testing, testing was required by 83% under one or more circumstances, such as upon arrival for the semester or ongoing surveillance testing. Among IHEs learning ≥50% in person, those with testing requirements were significantly more likely to have over 10,000 students compared to those with under 1000 students (Table 6). Testing requirements were more common in the Northeast than in the Midwest, and in urban than suburban areas.

**Table 6:** Characteristics of IHEs^a^ learning ≥50% in person with specific COVID-19 testing requirements versus those without

**IHE Characteristics**	**Any COVID-19 Testing Requirements**						
	**Requires, n (%)**^b^	**Does not Require, n (%)**^b^	***p*-value**	**Multivariable Analysis**^c^			
				**AOR**	**95% Confidence Limits**		***p*-value**
					**Lower**	**Upper**	
All IHEs offering ≥50% courses in person	138 (58)	89 (42)					
Affiliation							
Public	83 (64)	46 (36)	0.143	0.62	0.30	1.29	0.197
Private	55 (56)	43 (44)	–	1 (Referent)	–	–	–
Enrollment size^d^							
Under 1000 students	11 (35)	16 (65)	–	1 (Referent)	–	–	–
1000–4999 students	60 (60)	40 (40)	0.710	2.83	1.06	7.61	0.238
5000–9999 students	26 (63)	14 (37)	0.951	4.02	1.02	15.89	0.931
10,000–19,999 students	21 (73)	12 (27)	0.062	**7.49**	2.42	23.14	**0.027**
20,000 students and above	19 (77)	7 (23)	**0.037**	**10.35**	2.73	39.22	**0.012**
United States Census Region							
Northeast	46 (77)	10 (23)	–	1 (Referent)	–	–	–
Midwest	38 (48)	41 (52)	**0.043**	**0.19**	0.07	0.53	**0.024**
South	36 (55)	30 (45)	0.414	0.25	0.08	0.75	0.253
West	18 (59)	8 (41)	0.873	0.31	0.09	1.07	0.785
Degree of urbanicity^e^							
Rural	11 (81)	4 (19)	0.075	2.19	0.49	9.83	0.126
Town	40 (58)	27 (42)	0.607	1.08	0.43	2.71	0.743
Suburban	26 (45)	18 (55)	**0.029**	**0.37**	0.14	0.93	**0.004**
Urban	61 (60)	40 (40)	–	1 (Referent)	–	–	–
**IHE Characteristics**	**Requires COVID-19 Testing Upon Arrival for the Semester**						
	**Requires, n (%)**^b^	**Does not Require, n (%)**^b^	***p*-value**	**Multivariable Analysis**^c^			
				**AOR**	**95% Confidence Limits**		***p*-value**
					**Lower**	**Upper**	
All IHEs offering ≥50% courses in person	113 (47)	114 (53)					
Affiliation							
Public	69 (53)	60 (47)	0.156	1.16	0.50	2.70	0.735
Private	44 (45)	54 (55)	–	1 (Referent)	–	–	–
Enrollment size^d^							
Under 1000 students	10 (31)	17 (68)	–	1 (Referent)	–	–	–
1000–4999 students	54 (53)	47 (47)	0.140	2.49	0.90	6.92	0.345
5000–9999 students	21 (47)	19 (53)	0.738	1.98	0.53	7.39	0.849
10,000–19,999 students	14 (38)	19 (62)	0.470	1.30	0.26	6.51	0.484
20,000 students and above	14 (53)	12 (47)	0.386	3.26	0.61	17.39	0.271
United States Census Region							
Northeast	43 (70)	13 (30)	–	1 (Referent)	–	–	–
Midwest	29 (37)	50 (63)	0.063	0.26	0.10	0.64	0.094
South	26 (39)	40 (61)	0.128	0.26	0.10	0.70	0.128
West	15 (49)	11 (51)	0.961	0.40	0.13	1.27	0.979
Degree of urbanicity^e^							
Rural	10 (78)	5 (22)	**0.030**	3.09	0.75	12.69	0.111
Town	35 (52)	32 (48)	0.649	1.41	0.60	3.32	0.979
Suburban	26 (45)	18 (55)	0.201	0.87	0.35	2.13	0.131
Urban	42 (42)	59 (59)	–	1 (Referent)	–	–	–
**IHE Characteristics**	**Requires Surveillance Testing for COVID-19**						
	**Requires, n (%)**^b^	**Does not Require, n (%)**^b^	***p*-value**	**Multivariable Analysis**^c^			
				**AOR**	**95% Confidence Limits**		***p*-value**
					**Lower**	**Upper**	
All IHEs offering ≥50% courses in person	80 (33)	147 (67)					
Affiliation							
Public	49 (38)	80 (62)	0.213	**0.42**	0.20	0.87	**0.020**
Private	31 (31)	67 (69)	–	1 (Referent)	–	–	–
Enrollment size^d^							
Under 1000 students	4 (10)	23 (90)	–	1 (Referent)	–	–	–
1000–4999 students	31 (31)	70 (69)	0.214	**5.14**	1.23	21.43	**0.025**
5000–9999 students	17 (46)	23 (54)	0.358	15.10	2.78	81.88	0.369
10,000–19,999 students	14 (57)	19 (43)	**0.014**	**34.80**	7.39	163.89	**0.001**
20,000 students and above	14 (60)	12 (40)	**0.002**	**46.46**	8.07	267.42	**0.001**
United States Census Region							
Northeast	36 (57)	10 (43)	–	1 (Referent)	–	–	–
Midwest	15 (16)	64 (84)	**0.001**	**0.11**	0.04	0.31	**0.001**
South	20 (35)	46 (65)	0.913	0.32	0.11	0.92	0.924
West	9 (34)	17 (66)	0.968	0.35	0.10	1.19	0.903
Degree of urbanicity^e^							
Rural	7 (53)	8 (47)	0.121	**3.20**	0.92	11.11	**0.035**
Town	19 (22)	48 (78)	0.016	**0.73**	0.30	1.77	**0.046**
Suburban	21 (41)	23 (59)	0.524	1.28	0.46	3.56	0.937
Urban	33 (32)	68 (68)	–	1 (Referent)	–	–	–

^a^ IHEs, institutions of higher education

^b^ The universe of 1181 private IHEs was stratified by enrollment size. Based on sample size calculations using a 95% confidence interval with a 5% margin of error, 26% of eligible private IHEs were randomly selected within each stratum (< 1000: 97 of 398 private IHEs, 1000 – 4999: 161 of 625 private IHEs, 5000 – 9999: 24 of 93 private IHEs, 10,000 – 19,999: 12 of 47 private IHEs, and ≥ 20,000: 6 of 18 private IHEs). Weights represent the inverse of the sampling fractions. n (%) represents the unweighted number (n) and the weighted percent of the universe of 547 public and 1181 private IHEs

^c^ Multivariable logistic regression analysis of IHE characteristics with specific COVID-19 testing requirements as the dependent variable (0 = Does not require testing, 1 = Requires testing)

^d^ Enrollment size based on total students enrolled for credit as reported by the Integrated Postsecondary Education Data System

^e^ Level of urbanization based on urban boundaries by Census GEO as reported by the Integrated Postsecondary Education Data System

### Facemask policies

Facemasks were required by 88% of IHEs when indoors on campus property (Table 3). Similarly, 82% of IHEs required facemasks outdoors whenever a 6-ft distance could not be maintained. Policies requiring facemasks were least prevalent both indoors and outdoors in IHEs with < 1000 students.

## Discussion

### Main findings

While 96% of sampled IHEs deployed NPIs during the 2020–2021 academic year in response to the ongoing COVID-19 pandemic, only 1 in 5 (20%) comprehensively complied with the CDC guidance. Degree of compliance was associated with both IHE enrollment size and location, with larger IHEs (≥1000 students) and those located in the Northeast (Connecticut, Maine, Massachusetts, New Hampshire, New Jersey, New York, Pennsylvania, Rhode Island, and Vermont) being most likely to have high compliance. Conversely, the lowest compliance was most often observed in private IHEs and those located in the West (Arizona, Alaska, California, Colorado, Hawaii, Idaho, Nevada, New Mexico, Oregon, Washington, and Wyoming).

IHEs typically harbor high attack rates during outbreaks of respiratory illness; however, little comprehensive data on IHE outbreak response exist, including during the COVID-19 pandemic [19, 20]. Although measures introduced at the onset of the COVID-19 pandemic, such as distance learning and physical distancing, continue to be used [2, 21, 22], the scientific evidence base on their effects and effectiveness in reducing respiratory virus transmission is still evolving, and it was even more limited in early 2020 when the initial CDC IHE guidance was issued [11]. IHEs that re-opened for the 2020–2021 academic year reported implementation of a more streamlined set of NPIs across the US and globally, including distancing in the learning environment, use of facemasks, and COVID-19 testing protocols, which have been associated with decreased transmission of SARS-CoV-2, particularly in high-density settings such as IHEs [6, 8, 9, 23–28]. Much of the currently available literature is focused on the implementation of few or individual NPIs [25, 27–29]. For example, epidemiologic models have concluded that even 20% uptake of facemasks can significantly reduce epidemic size under a full IHE campus re-opening [24]. Additionally, surveillance testing of campus populations has been associated with lower transmission of SARS-CoV-2 than less frequent or symptomatic-only testing policies [26, 27]. While there is evidence that suggests distance-based interventions are not always associated with reduced transmission of SARS-CoV-2 [30], the literature frequently echoes the finding that physical distancing in combination with other NPIs more effectively prevents COVID-19 than any NPI alone [31, 32]. Classroom-specific interventions, such as hybrid instruction and limited class sizes, have been reported to substantially reduce the basic reproduction number in models of IHE-related COVID-19 transmission [33]. However, it is also shown that without dramatic reduction in class sizes or meeting frequency these decreases are not significant and will heavily depend on laboratory testing and other physical distancing measures [34, 35]. Although most currently available studies are limited to specific populations and not fully generalizable to IHEs across the US [6, 8, 9, 23], they suggest that regardless of enrollment size or location, IHEs must take a multifaceted approach and timely implement combinations of appropriate NPIs to effectively reduce transmission of SARS-CoV-2 on campus [9, 31, 32, 34, 35].

Among IHEs where the primary learning method was identified, about one-third reported offering classes primarily through distance education. Our data suggest that large or public IHEs were more likely to have primarily offered distance learning, while also being more likely to have made physical changes to the learning environment when utilizing in-person instruction. Modalities of learning environment changes such as distanced seating configurations, hybrid schedules, and reduced class sizes are widely used to maximize in-person opportunities while minimizing the risk of SARS-CoV-2 transmission by decreasing student contact [34–38]. Notably, distance learning has been reported to create significant challenges for students, particularly those of low- and middle-income backgrounds or those who rely on on-campus facilities and services for accessible technology, quiet space, or stable housing [39–42]. Reasons for preference in learning method are complex and are beyond the scope of this research; however, factors such as logistic capabilities, funding, local politics, and perceptions of distance learning may have played roles in decision making [2, 43, 44]. Therefore, IHEs should carefully consider the benefits of in-person operations, their ability to implement NPIs to facilitate safe in-person operations, and the health of IHE populations and risks for surrounding communities during a severe pandemic such as COVID-19 [28, 45].

Two-thirds of IHEs made changes to the academic calendar in response to COVID-19. Lengthening of breaks, either by transitioning to a period of distance learning or by adjusting academic start or end dates, acts as a temporary IHE dismissal allowing for longer periods between mixing of campus populations with outside populations and re-congregation on campus. These have been employed both proactively and reactively by IHEs to reduce campus-based SARS-CoV-2 transmission [6, 8, 23]. Elimination of breaks – a measure that is not a part of CDC pre-pandemic guidelines or pandemic guidance – reduces the potential number of consecutive student days away from campus in the middle of the term, theoretically reducing opportunities to bring new viral lineages onto IHE campuses and surrounding communities [29]. For example, a large cluster of COVID-19 cases on a Chicago university campus following spring break revealed roughly two-thirds of sequenced cases originated outside of Chicago [46]. It is unlikely, however, that the break elimination would reduce already established virus transmission.

Despite widespread efforts to reduce density and limit visitation as IHEs re-opened campus for the 2020–2021 academic year, many reported COVID-19 outbreaks linked to residential housing due to the difficulty of maintaining and enforcing physical distancing measures in those spaces [8, 23, 27, 47, 48]. IHE policies restricting congregation in campus spaces and housing may be difficult to enforce and do not extend to off-campus student gatherings.

Our data suggest that larger IHEs were more likely than smaller IHEs to require COVID-19 testing. On-campus surveillance testing tends to be the most expensive and logistically demanding COVID-19 mitigation measure, and frequent deployment may pose a challenge, particularly to small IHEs, which receive proportionally less enrollment-based funding or may lack extensive laboratory infrastructure [26, 43, 49]. Because testing is an important public health tool to reduce SARS-CoV2 transmission in dense congregate settings such as IHEs, reasons for limited compliance with testing recommendations should be elucidated and addressed [27, 50, 51].

Compared to laboratory testing, facemasks pose less of a financial and logistical burden on IHEs [44]. A majority of IHEs required facemasks indoors and outdoors wherever six-foot spacing could not be maintained. The encouraged or required use of facemasks has been consistently cited as a core NPI utilized by IHEs in response to COVID-19 [23, 24, 33, 52]. Observational reports reveal high levels of compliance with facemask mandates on IHE campuses [25], and they remain an important measure for reducing SARS-CoV2 transmission, especially in dense indoor settings.

### Limitations

Our results should be considered in the context of at least five limitations. First, publicly available data are not guaranteed to fully reflect all NPIs implemented within IHEs. However, our method allowed us to obtain the data on recommended NPIs quickly from a wide variety of measures from a large sample of public and private institutions. Additionally, the CDC Guidance for IHEs made broad recommendations intended to be applied by a wide variety of IHEs; as such, it left room for interpretation in how IHEs implemented the guidance, which in turn posed a challenge in the data collection process. If an NPI was not explicitly mentioned on an IHE website, it was recorded as “not found,” minimizing assumptions and misclassification biases. Public IHEs were more likely to be larger and have publicly available COVID-19 information than private IHEs, which may contribute to the enrollment size- or affiliation-related variations in documented NPIs.

Second, a team of several people was responsible for data collection, which can lead to systematic bias. Response frequencies for each NPI were compared within and between each member of the data collection team to ensure patterns or outliers were consistent with the original data sources. Acceptable patterns were found to be due to university systems or state and local governments issuing streamlined guidance for all IHEs under their influence.

Third, data were collected in the framework of a cross-sectional study, whereby information on NPIs for public IHEs were collected from December 2020–May 2021 and for private IHEs were collected from April 2021–June 2021. The difference in data collection periods between public and private IHEs may have introduced bias, particularly with regard to evolving policies in an active pandemic. Additionally, because data collection occurred during the second semester of the 2020–2021 academic year, changes made during the first semester may have been missed. Although this has the potential to introduce response bias as publicly available information evolves over time, data were collected systematically, ensuring the data described measures representing NPIs actively implemented or how they evolved throughout the entire 2020–2021 academic year.

Fourth, due to the impermanent nature of webpages, the data collected during the study period may no longer be publicly available or may only exist in archive. We have included IHE webpage addresses and dates of data collection to specify data location and timing.

Finally, this study aims to evaluate and describe the steps taken by IHEs to minimize SARS-CoV-2 transmission on campus and does not account for adherence to the documented NPIs, or off-campus behavior.

## Conclusions

In conclusion, the COVID-19 pandemic had an unprecedented impact on IHEs across the US, where there has previously been little comprehensive data on respiratory illness outbreaks and responses. Some NPIs were widely implemented as a means of reducing SARS-CoV2 transmission within IHEs, but the degree of compliance with recommended NPIs varied by IHE enrollment size and location. Further research is needed to understand the reasons for suboptimal compliance, including the interactions of economic, logistic, political, and socio-behavioral factors, to understand the relationship of implementation across multiple NPIs, to explore the role of urbanization and dynamics of the surrounding community, and to address barriers to the implementation of recommended NPIs. In future studies evaluating the effectiveness of these measures in IHE settings, level of compliance with NPIs as described here, as well as levels of adherence and the impact of off-campus behaviors should be taken into consideration. As IHEs continue to navigate the ongoing COVID-19 pandemic, they must adapt their normal operations to prioritize the health of students and staff through layered COVID-19 prevention, including vaccination, timely case detection through testing and tracing, and continued use of NPIs as feasible and appropriate for the local epidemiologic situation. In addition to the discussed NPIs, for sustainable control of respiratory infections, including COVID-19, IHEs are encouraged to improve campus ventilation infrastructure, increase opportunities for physical distancing such as open-air study spaces, exercise flexibility in distance learning and staying home when sick, and promote the consistent use of facemasks during the seasonal waves of respiratory infections, particularly for anyone (students, staff, visitors) who is experiencing respiratory symptoms [10].

## Supplementary Information

**Additional file 1.**

## Data Availability

All data generated or analyzed during this study are included in this published article and its additional information files or available from public sources identified in the paper. The list of sampled IHEs, abstracted and analyzed data, and the corresponding codebook of variable definitions are available in Additional file 1. CDC Guidance for IHEs can be accessed at https://www.cdc.gov/coronavirus/2019-ncov/community/colleges-universities/considerations.html and http://web.archive.org/web/20201029222917/https://www.cdc.gov/coronavirus/2019-ncov/community/colleges-universities/considerations.html. The National Center for Education Statistics Integrated Postsecondary Education Data System can be accessed at https://nces.ed.gov/ipeds. US Census Regions and population estimates are available from https://www.census.gov/geographies/reference-maps/2010/geo/2010-census-regions-and-divisions-of-the-united-states.html and https://www.census.gov/programs-surveys/popest/technical-documentation/research/evaluation-estimates/2020-evaluation-estimates/2010s-counties-total.html. COVID-19 case surveillance data was obtained from https://github.com/CSSEGISandData/COVID-19. For questions regarding the study method and data collection, and the data you are welcome to contact the corresponding author.

## Abbreviations

NPIs: Nonpharmaceutical interventions
US: United States
SARS CoV-2: Severe acute respiratory syndrome coronavirus 2
COVID-19: Coronavirus disease 2019
IHEs: Institutions of higher education
CDC: US Centers for Disease Control and Prevention
NCES: National Center for Education Statistic
IPEDS: Integrated Postsecondary Education Data System
CI: National COVID-19 incidence rate per 100,000 population
